# Effectiveness of Regional Blocks for Postoperative Pain Control and Recovery in Breast Cancer Surgery in a Resource-Limited Setting: A Prospective Observational Study

**DOI:** 10.7759/cureus.98392

**Published:** 2025-12-03

**Authors:** Amila P Nellihela, Ruchika N Senevirathne, Vathsal Bandaranayake, Vishaka Kerner, Aruna Jayasena, Pirahanthan Karunanithy, Kasun A Rajapakshe, Sachini Weddagala, Sayomi Warnakula, Mahesh Senarathna

**Affiliations:** 1 Department of Surgical Oncology, Teaching Hospital Anuradhapura, Anuradhapura, LKA; 2 Department of Cardiology, Teaching Hospital Anuradhapura, Anuradhapura, LKA; 3 Department of Anesthesia and Critical Care and Pain Medicine, Teaching Hospital Anuradhapura, Anuradhapura, LKA; 4 Faculty of Medical Sciences, University of Sri Jayewardenepura, Sri Jayewardenepura, LKA; 5 Department of General Surgery, Royal Prince Alfred Hospital, Sydney, AUT; 6 Department of Anesthesia, Postgraduate Institute of Medicine, University of Colombo, Colombo, LKA; 7 Department of Anesthesiology, Teaching Hospital Peradeniya, Peradeniya, LKA; 8 Postgraduate Institute of Science, University of Peradeniya, Peradeniya, LKA

**Keywords:** breast cancer, chronic post-surgical pain, enhanced recovery after surgery (eras) protocols, opioid-sparing, postoperative nausea and vomiting (ponv), postoperative pain, regional anaesthesia, resource-limited setting, sri lanka, ultrasound-guided nerve blocks

## Abstract

Background and aims

Effective postoperative pain management after breast cancer surgery is essential to enhance recovery, minimise opioid use, and prevent chronic post-surgical pain. In low- and middle-income countries (LMICs) like Sri Lanka, limited access to ultrasound and trained personnel often restricts the use of regional anaesthesia (RA). Although regional anaesthesia (RA) is well-established for breast cancer surgery, evidence from low- and middle-income countries remains limited. This study evaluated the effectiveness and feasibility of ultrasound-guided regional nerve blocks in improving postoperative outcomes in a resource-limited healthcare setting.

Material and methods

A prospective observational study was conducted at the Surgical Oncology Unit, Teaching Hospital Anuradhapura, from October 2023 to February 2024. Fifty-six female patients undergoing major breast cancer surgery under general anaesthesia were allocated to receive either regional anaesthesia (paravertebral, erector spinae, or pectoral nerve block) or local wound infiltration. Forty-one (73.2%) received ultrasound-guided regional blocks (paravertebral (n = 16), erector spinae plane (ESP) (n = 13), or pectoral nerve (PECS I/II) blocks (n = 12)) and fifteen (26.8%) received local anaesthetic infiltration. Pain scores were assessed using the visual analogue scale (VAS) at defined postoperative intervals. Morphine consumption, postoperative nausea and vomiting (PONV), mobilisation time, and three-month chronic pain were compared using independent-samples t-tests and chi-square/Fisher’s exact tests, with p < 0.05 considered significant.

Results

Patients receiving regional anaesthesia (RA) reported significantly lower postoperative pain scores at 4 hours (2.5 ± 1.9 versus 4.7 ± 1.9; p = 0.001), 6 hours (1.9 ± 1.3 versus 3.2 ± 1.5; p = 0.003), and 12 hours (1.4 ± 0.8 versus 2.6 ± 1.5; p = 0.009) compared with the local infiltration group. By 24 hours, pain scores were low and comparable between groups. Mean morphine consumption was significantly lower in the RA group (2.6 ± 5.8 mg versus 7.5 ± 3.6 mg; p = 0.004), and 71% of RA patients required no opioids postoperatively. The incidence of postoperative nausea and vomiting (PONV) was also reduced (mean score: 1.2 ± 0.4 versus 1.7 ± 0.5; p < 0.001). Patients who received RA achieved earlier mobilisation (6.0 ± 1.5 hours versus 7.0 ± 1.4 hours; p = 0.032), and none developed chronic pain at three months, compared with 40% in the infiltration group (p = 0.009). No block-related complications were observed.

Conclusions

Regional anaesthesia techniques provided superior postoperative analgesia, markedly reduced opioid and antiemetic requirements, and facilitated earlier mobilisation following breast cancer surgery in a resource-limited setting. These blocks were feasible, safe, and highly effective despite infrastructural constraints. Incorporating regional anaesthesia into multimodal analgesia protocols in low- and middle-income countries may substantially improve recovery, enhance patient satisfaction, and reduce the burden of chronic post-mastectomy pain.

## Introduction

Breast cancer surgery is frequently associated with moderate to severe acute postoperative pain and carries a substantial risk of developing chronic post-mastectomy pain syndrome, which affects approximately 30%-50% of patients [1,2]. Inadequate management of acute postoperative pain not only increases immediate patient discomfort but is also a known risk factor for the transition to chronic pain, delayed mobilisation, prolonged hospital stay, and increased reliance on systemic opioids [2]. Consequently, optimal perioperative pain control is a key component of Enhanced Recovery After Surgery (ERAS) pathways in breast oncology [3].

Regional anaesthesia (RA) techniques, including thoracic paravertebral block (PVB), pectoral nerve blocks (PECS I and II), erector spinae plane (ESP) block, and serratus anterior plane block (SAPB), have become central to multimodal analgesia strategies in this setting [4,5]. PVB remains the reference standard for mastectomy analgesia due to robust evidence supporting its effectiveness in reducing both acute and chronic postoperative pain [6-8].

Despite this progress, RA uptake in breast cancer surgery remains limited in many low- and middle-income countries (LMICs), including Sri Lanka. Barriers include limited ultrasound availability, inadequate numbers of trained RA providers, and constrained theatre time [9,10]. Local studies have demonstrated that ultrasound-guided ESP and SAPB blocks are both feasible and effective for mastectomy analgesia in Sri Lankan settings [11-13], but their widespread implementation remains inconsistent. The COVID-19 pandemic further exacerbated these barriers by reducing theatre capacity and anaesthesia staffing allocation [10]. As a result, general anaesthesia combined with surgeon-delivered local anaesthetic infiltration and systemic opioids continues to be the dominant analgesic strategy in Sri Lanka. While practical, this approach often provides only short-lived postoperative analgesia and may predispose patients to rebound pain and increased opioid use later in recovery [12,13].

Accordingly, we conducted a prospective observational study at a tertiary centre in Sri Lanka to compare the analgesic efficacy and recovery outcomes of regional anaesthesia techniques versus local infiltration in patients undergoing mastectomy. The primary objective was to evaluate differences in postoperative pain scores and opioid consumption between the two approaches. Secondary objectives were to compare the incidence of postoperative nausea and vomiting (PONV), early mobilisation, and chronic pain at three months. In addition, a prespecified objective was to assess the feasibility of incorporating ultrasound-guided regional anaesthesia into routine breast cancer surgery in this resource-limited setting, as reflected by the proportion of eligible patients receiving blocks, practical barriers to their use, and the occurrence of any block-related complications.

## Materials and methods

Study design and setting

This prospective observational study was conducted at the Surgical Oncology Unit in collaboration with the Department of Anaesthesia of Teaching Hospital Anuradhapura, Sri Lanka, from October 2023 to February 2024. The hospital is a tertiary-level oncology referral centre serving a large regional population. Ethical approval was obtained from the Ethics Review Committee of the Faculty of Medicine and Allied Sciences, Rajarata University of Sri Lanka (ERC/2024/76). Institutional review board (IRB) clearance was obtained from the Director, Teaching Hospital Anuradhapura, Sri Lanka. Written informed consent was obtained from all patients prior to participation, and the study adhered to the principles of the Declaration of Helsinki.

Study population and inclusion and exclusion criteria

The study population consisted of adult female patients diagnosed with breast cancer and scheduled to undergo major breast surgery under general anaesthesia. Inclusion criteria were as follows: female sex, age ≥ 18 years, and undergoing modified radical mastectomy, simple mastectomy, wide local excision with axillary clearance, or mastectomy with immediate reconstruction. Exclusion criteria were as follows: surgeries for benign breast disease such as fibroadenoma excision, minor day-case breast procedures, previous ipsilateral thoracic surgery or pre-existing chronic pain disorders, and contraindications to regional anaesthesia such as infection at the injection site or coagulopathy.

Sampling and group allocation

A total of 56 consecutive eligible patients were enrolled. Group allocation was non-randomised and followed real-world service conditions. Patients received regional anaesthesia (RA) when ultrasound equipment and a trained anaesthetist were available prior to induction. When these resources were not available, patients received local anaesthetic infiltration by the surgical team. This pragmatic allocation reflected standard clinical workflow in a resource-limited environment and allowed assessment of RA feasibility in routine practice [14].

Anaesthesia and analgesia protocol

All procedures were performed under standard general anaesthesia induced with intravenous propofol and a neuromuscular blocking agent, and maintained with volatile anaesthetic in an oxygen/air mixture. All patients received a uniform anaesthetic regimen. No NSAIDs, ketamine, dexmedetomidine, magnesium, or other analgesic adjuvants were used. Review of anaesthetic charts confirmed that this protocol was consistently applied across both groups, eliminating confounding from intraoperative opioid or adjuvant variability. Patients in the RA group received a unilateral ultrasound-guided thoracic paravertebral block, erector spinae plane block, or pectoral nerve (PECS I/II) block prior to surgical incision, using 20-25 mL of 0.25%-0.5% bupivacaine. Block choice was based on anatomical exposure, anticipated surgical field, and operator expertise. When feasible, sensory loss to pinprick was assessed to confirm block success. Patients in the local infiltration group received 20 mL of 0.25% bupivacaine with adrenaline 1:200,000 infiltrated into the surgical wound bed during closure [15].

Postoperative analgesia followed a standard protocol across all patients. Paracetamol was administered regularly, and NSAIDs were prescribed as clinically appropriate. Patient-controlled analgesia (PCA) pumps were minimally available in our unit, with only a single device accessible on the ward during the study period. Consequently, only a small proportion of patients received PCA morphine. For those who did, the PCA was set to deliver 1 mg morphine boluses with a five-minute lockout interval and no background infusion. The majority of patients instead received intermittent intravenous morphine boluses administered by nursing staff or low-dose morphine infusions when PCA was unavailable. All opioid delivery routes, including PCA doses, nurse-administered boluses, and infusions, were recorded and incorporated into the 24-hour cumulative morphine consumption, which served as the postoperative opioid outcome measure.

**Figure 1:**
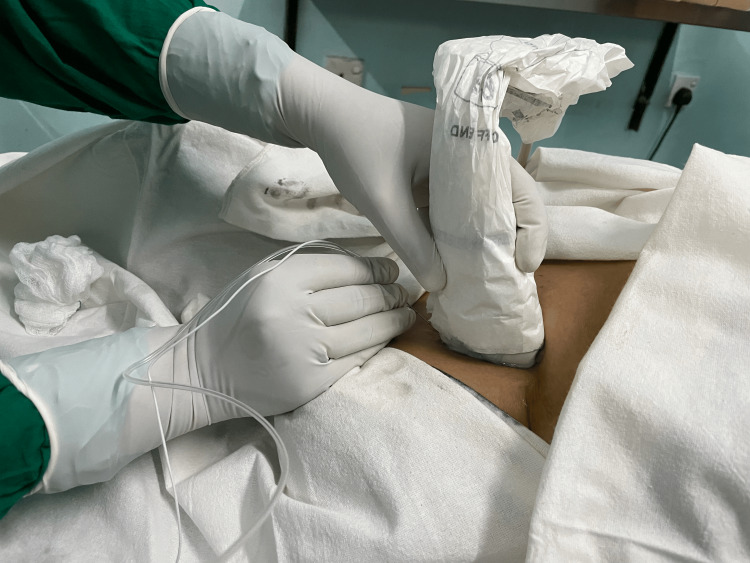
Ultrasound-guided PECS I/II block being administered PECS: pectoral nerve block

**Figure 2:**
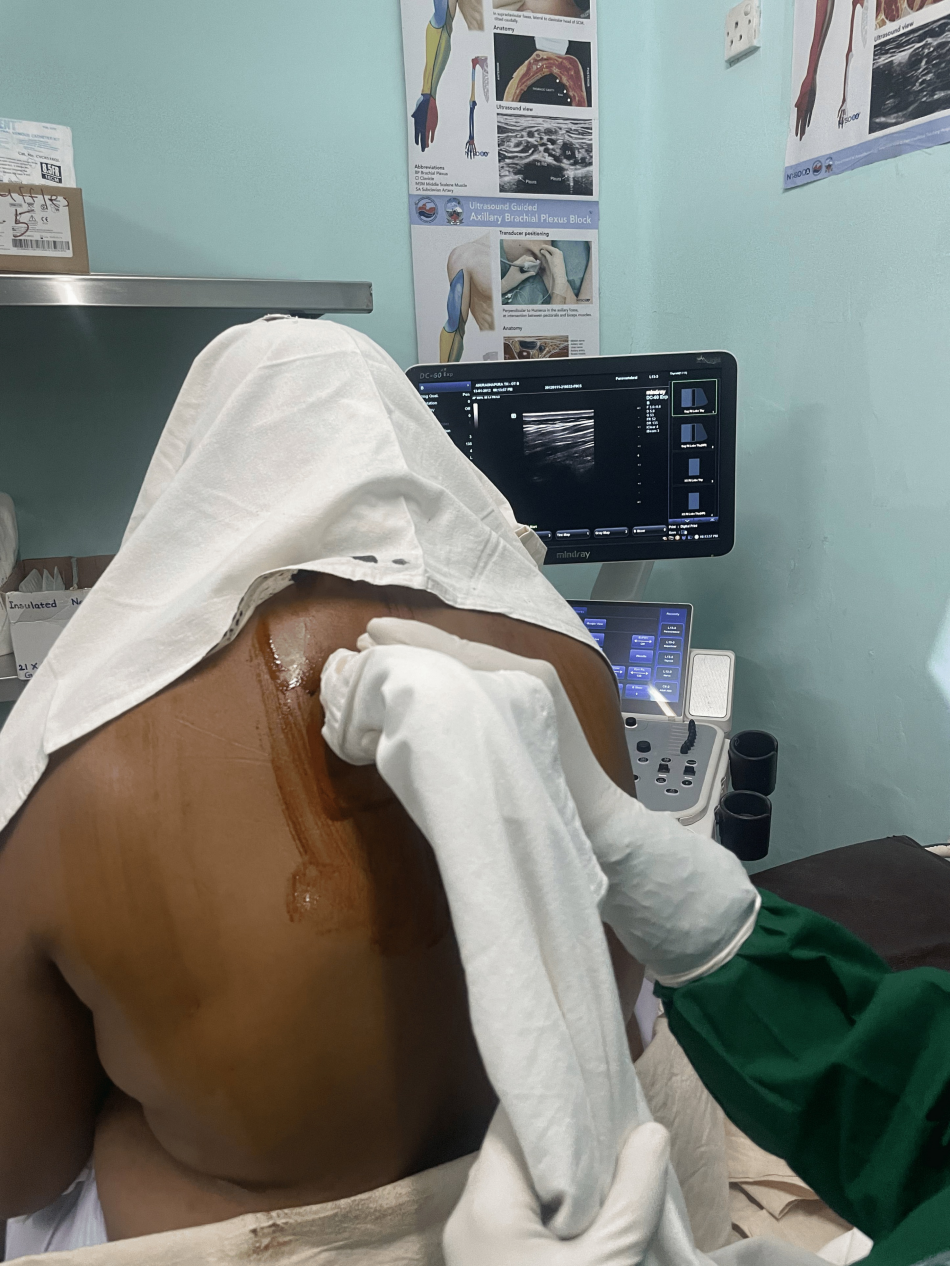
Ultrasound-guided paravertebral block being administered

**Figure 3:**
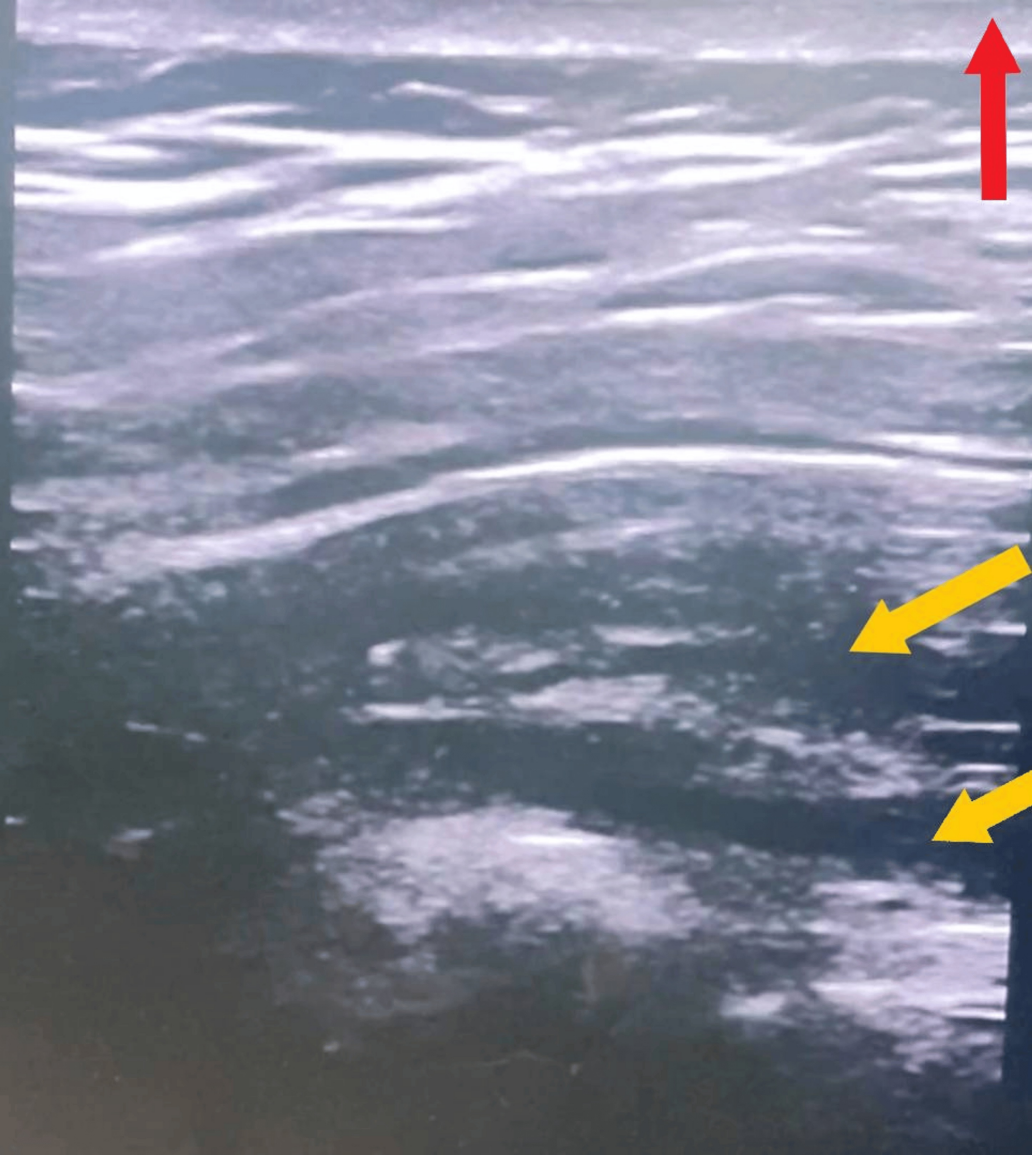
Ultrasound view of the paravertebral block being administered Yellow arrows show the local aesthetic injected expanding between tissue planes. The red arrow points to the skin surface.

The regional techniques employed in this study are illustrated in Figures 1-3. Figure 1 demonstrates the ultrasound-guided PECS I/II block with clear identification of the pectoral fascial planes. Figure 2 shows the needle approach for the paravertebral block under real-time ultrasound guidance. Figure 3 provides a transverse ultrasound image of the paravertebral space with spatial orientation markers indicating the skin surface (red arrow) and local anaesthetic spread between tissue planes (yellow arrows). These figures are included to enhance transparency of block performance, facilitate reproducibility, and meet journal requirements for explicit spatial orientation markers.

Outcome measures

The primary outcome measure was acute postoperative pain intensity, assessed using a 10-point visual analogue scale (VAS; 0 = no pain, 10 = worst pain imaginable). Pain scores were recorded at 4, 6, 12, and 24 hours after surgery by trained nursing staff. These time points were selected to capture the early postoperative pain trajectory: the 4-12-hour period corresponds to the expected duration of single-shot regional blocks, while the 24-hour assessment reflects pain levels after block resolution and standard analgesics have taken effect [16].

Secondary outcome measures evaluated opioid requirements, side effects, functional recovery, and longer-term pain outcomes. These included the following: (1) total morphine consumption within the first 24 hours postoperatively (mg); (2) incidence of postoperative nausea and vomiting (PONV) within 24 hours, assessed using a three-point scale (0 = no nausea/vomiting, 1 = nausea only, 2 = vomiting requiring treatment); (3) time to mobilisation, defined as the time from completion of surgery to first sitting upright in bed and first ambulation with assistance (hours); and (4) presence of chronic pain at three months, defined as any persisting pain at the surgical site with VAS ≥ 3 during routine follow-up, consistent with established definitions of chronic post-surgical pain [15,16].

Mobilisation protocol

Postoperative mobilisation followed a standardised ERAS-aligned institutional protocol used for all major breast surgeries. Upon arrival to the postoperative ward, patients were assessed by nursing staff to ensure they were awake, haemodynamically stable, and not experiencing significant nausea or dizziness. Mobilisation consisted of the following: (1) sitting upright at the edge of the bed between four and six hours post-surgery and (2) first ambulation with assistance between 8 and 12 hours postoperatively, supervised by a nurse or physiotherapy assistant, with accommodations made for surgical drains. Patients were also encouraged to perform deep-breathing exercises and gentle shoulder mobilisation. This protocol was applied uniformly to both study groups.

Sample size determination and power analysis

The sample size was determined based on the primary endpoint of early postoperative pain reduction. Published comparative studies demonstrate that regional nerve blocks typically reduce postoperative pain by 1.0-1.5 VAS points, with a standard deviation (SD) of approximately 2.0 points [17-20]. Assuming a clinically meaningful difference of 1.5 points between groups, a two-tailed α of 0.05, and 80% power, the minimum required sample size was ~30 patients per group using the standard two-sample comparison framework [21]. The assumed difference exceeds the minimal clinically important difference for acute pain on a 100-mm VAS (≈12-13 mm) [22]. In the present study, operational feasibility resulted in 41 patients receiving regional anaesthesia and 15 patients receiving local infiltration. A post hoc power analysis confirmed that the achieved sample provided >80% power to detect a 2-point difference in VAS scores (SD ≈ 2), supporting adequacy of power for the primary outcome.

Data collection and statistical analysis

Clinical and perioperative data were collected using structured proformas by personnel not involved in block administration. Pain and PONV assessments were recorded by nursing staff independent of group allocation. Chronic pain assessments were performed at routine three-month postoperative oncology follow-up.

Data analysis was conducted using IBM SPSS Statistics version 25 (IBM Corp., Armonk, NY). We first conducted descriptive analyses of the study cohort. Continuous variables are presented as mean ± standard deviation (SD), and categorical variables are summarised as counts and percentages. For comparisons between the regional block group and local infiltration group, we used independent-samples t-tests for continuous data (e.g., pain scores and morphine amounts) when approximately normally distributed. Categorical data (e.g., presence of PONV and incidence of chronic pain) were compared using chi-square tests or Fisher’s exact tests when cell counts were small. A two-tailed p-value < 0.05 was considered statistically significant for all analyses.

## Results

Patient characteristics and group allocation

Fifty-six patients met the inclusion criteria. Of these, 41 (73%) received a regional anaesthesia technique and 15 (27%) received local wound infiltration. Among the regional anaesthesia group, paravertebral block was performed in 16 (39%) patients, erector spinae plane block in 13 (32%) patients, and PECS I/II block in 12 (29%) patients. The age distribution, surgical procedure type, and other baseline characteristics were similar between groups. No immediate block-related complications were recorded.

Acute postoperative pain

**Figure 4:**
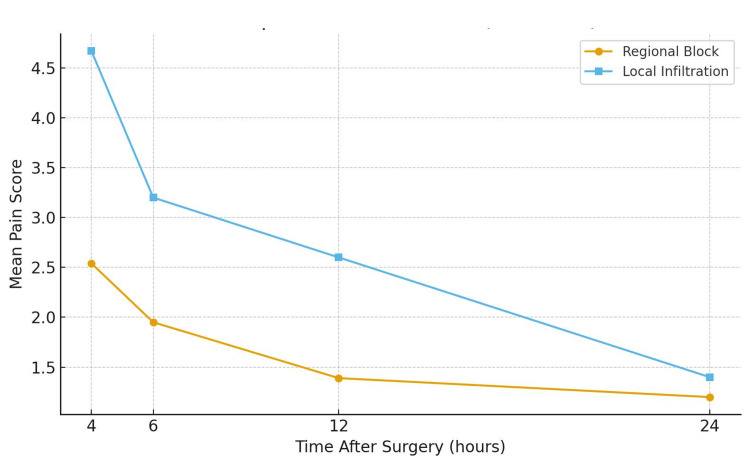
Mean VAS (VAS 0-10) pain scores at 4, 6, 12, and 24 hours postoperatively for the regional anaesthesia and local infiltration groups VAS: visual analogue scale

Mean VAS scores at each postoperative time point are shown in Figure 4. At four hours, the mean pain score was 2.54 ± 1.91 in the regional anaesthesia group and 4.67 ± 1.92 in the infiltration group (p = 0.001). At six hours, scores were 1.95 ± 1.28 and 3.20 ± 1.47, respectively (p = 0.003). At 12 hours, scores were 1.39 ± 0.80 and 2.60 ± 1.50 (p = 0.009). At 24 hours, scores were 1.20 ± 1.47 and 1.40 ± 0.63 (p = 0.60).

Opioid Consumption

**Figure 5:**
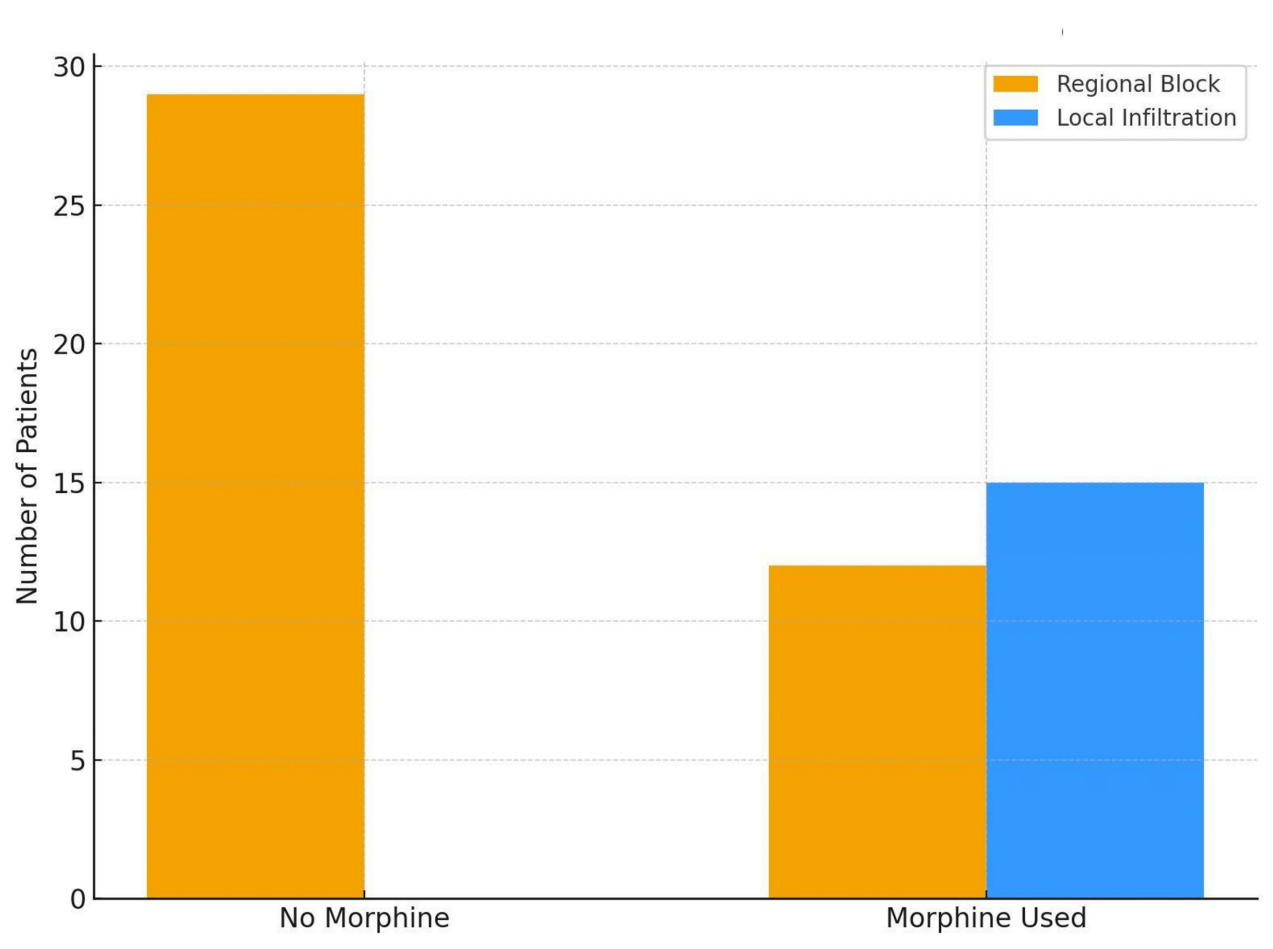
Bar chart showing the number of patients in the regional anaesthesia and local infiltration groups who received postoperative morphine within the first 24 hours after surgery

Cumulative morphine use within 24 hours is summarised in Figure 5. Mean morphine consumption was 2.6 ± 5.8 mg in the regional anaesthesia group and 7.5 ± 3.6 mg in the infiltration group (p = 0.004). In the regional anaesthesia group, 29 of 41 (70.7%) patients required no morphine, while all 15 patients in the infiltration group received morphine.

Postoperative Nausea and Vomiting

**Figure 6:**
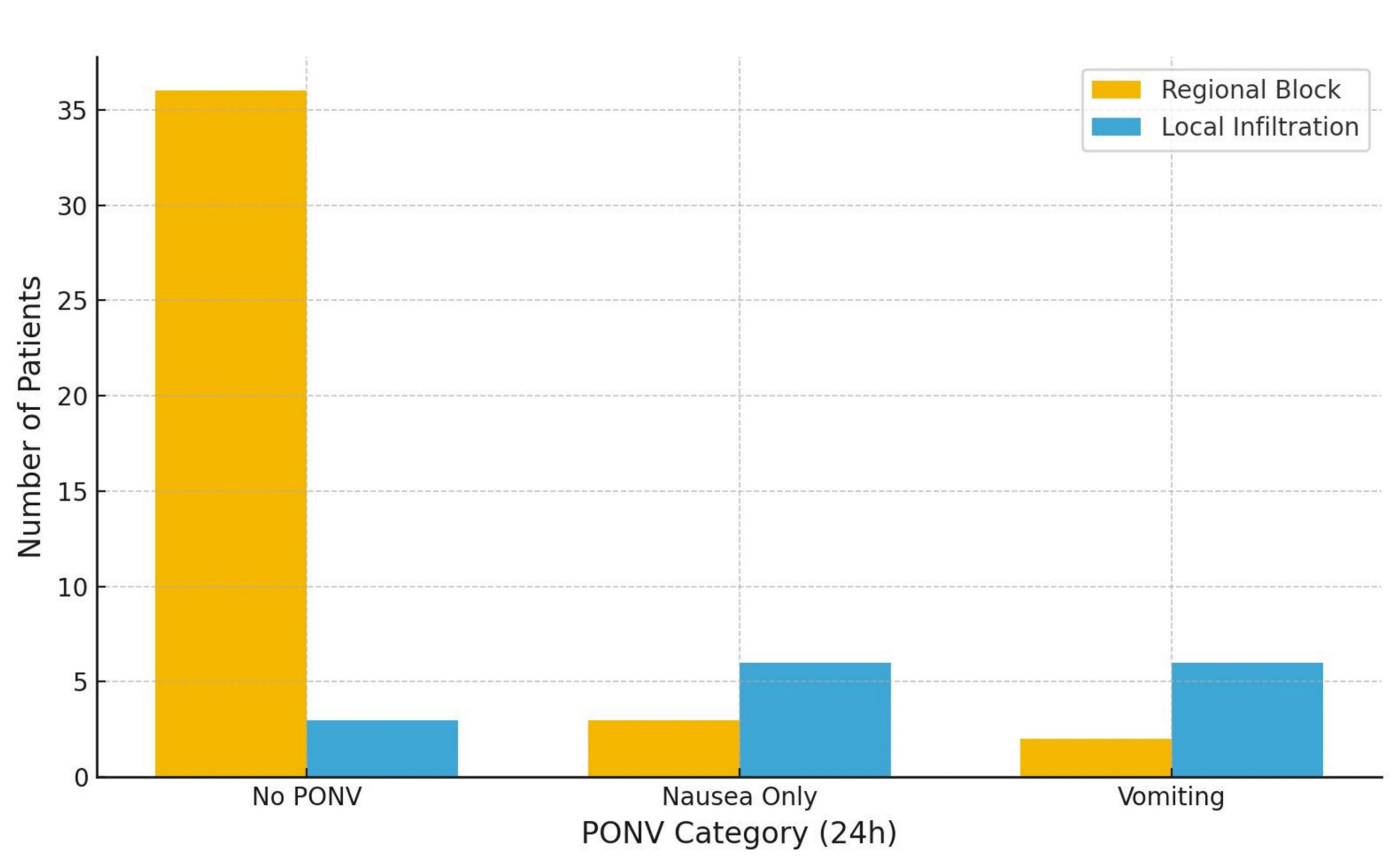
Bar chart showing the proportions of patients with no PONV, nausea only, or vomiting requiring treatment in the regional anaesthesia and local infiltration groups within the first 24 hours after surgery PONV: postoperative nausea and vomiting

Figure 6 presents the distribution of PONV scores. In the regional anaesthesia group, 36 (87.8%) patients had no PONV, 3 (7.3%) patients experienced nausea, and 2 (4.9%) patients experienced vomiting requiring treatment. In the infiltration group, 3 (20%) patients had no PONV, 6 (40%) patients had nausea, and 6 (40%) patients had vomiting. Mean PONV scores were 1.20 ± 0.40 and 1.73 ± 0.46, respectively (p < 0.001).

Time to mobilisation

**Figure 7:**
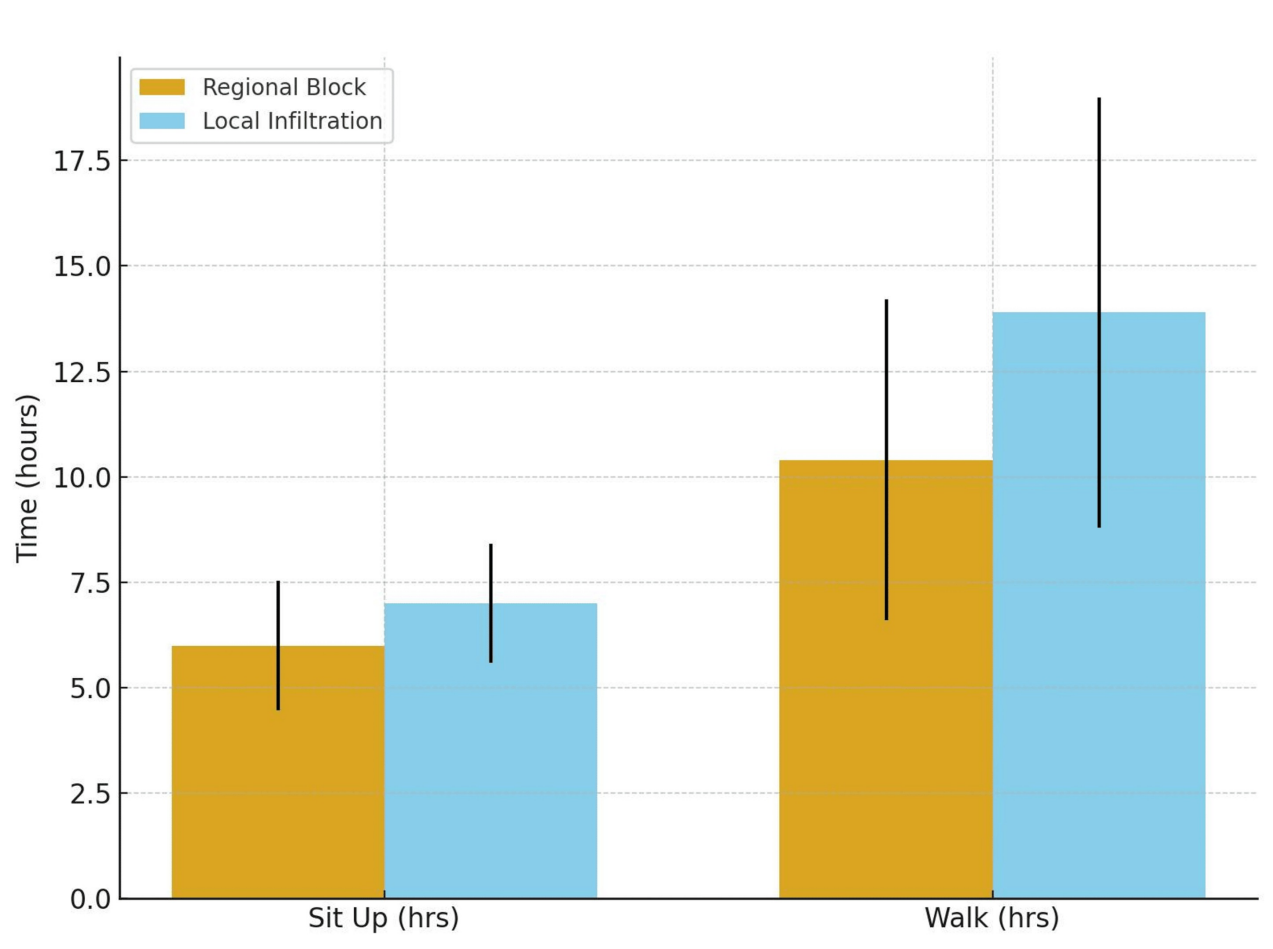
Time to mobilisation by group mean (± SD) time to first mobilisation (sitting upright) and first ambulation (walking with assistance), in hours, for the regional anaesthesia and local infiltration groups Error bars represent SD. SD: standard deviation

Mobilisation times are shown in Figure 7. Mean time to first sitting upright was 6.0 ± 1.53 hours in the regional anaesthesia group and 7.0 ± 1.41 hours in the infiltration group (p = 0.032). Mean time to first ambulation was 10.4 ± 3.8 hours and 13.9 ± 5.1 hours, respectively (p ≈ 0.07).

Chronic Pain at Three Months

At three-month follow-up, chronic pain was reported by 0 of 41 (0%) patients in the regional anaesthesia group and 6 of 15 (40%) patients in the infiltration group (p = 0.009). Reported pain scores in affected patients ranged from VAS 3 to 5.

## Discussion

This prospective observational study found that the use of regional anaesthesia (RA) was associated with lower postoperative pain scores, reduced opioid requirements, fewer episodes of postoperative nausea and vomiting (PONV), and earlier mobilisation among patients undergoing breast cancer surgery. These associations align with the existing evidence base supporting RA in breast surgery and illustrate its potential applicability within resource-limited settings [7].

Acute pain management and opioid-sparing effects

A substantial body of literature confirms that RA reduces postoperative pain, opioid requirements, and opioid-related side effects such as PONV [4]. Early and targeted regional analgesia may also attenuate central sensitisation and reduce the risk of chronic post-surgical pain [5]. PECS and ESP blocks, in particular, have gained popularity as simpler and faster alternatives to paravertebral blocks, making them attractive options in high-volume surgical services. These blocks are increasingly incorporated into ERAS programmes as effective components of multimodal analgesia without compromising analgesic outcomes [3].

Randomised controlled trials and meta-analyses continue to show that RA techniques provide superior analgesia compared with systemic analgesia or local anaesthetic infiltration [4]. Previous work reports that PECS II blocks may reduce the incidence of chronic pain [7], while ESP blocks provide significant opioid-sparing effects [8]. In our study, patients who received RA (paravertebral, erector spinae, or pectoral nerve blocks) reported significantly lower postoperative pain scores than those who received only local infiltration [8]. The greatest differences were observed in the first 12 hours, aligning with the expected duration of single-shot blocks. A 1.5-2 point reduction on the 10-point VAS represents a clinically meaningful improvement in comfort, consistent with other literature demonstrating the superiority of RA over infiltration techniques [15,17].

The broader dermatomal coverage and more sustained analgesic effect offered by blocks such as PVB, ESP, and PECS likely explain their advantage over local infiltration, which typically provides only short-lived relief. Sri Lankan data demonstrating improved postoperative outcomes following structured analgesia programmes further support the rationale for integrating RA into perioperative care pathways [23,24].

Our study also demonstrated a substantial opioid-sparing effect. RA patients used approximately one-third of the morphine consumed by the infiltration group, and over 70% remained completely opioid-free within 24 hours. This reduction has significant clinical implications, as minimising opioid exposure reduces risks of sedation, respiratory depression, and PONV. Consistent with this, our RA group experienced markedly lower PONV rates, consistent with findings from multimodal RA-based analgesia pathways [8,25]. Improved PONV outcomes contribute meaningfully to patient comfort and may support earlier oral intake and mobilisation.

Chronic pain prevention

One of the most notable findings of our study was the absence of chronic post-mastectomy pain at three months in the RA group compared to a 40% incidence in the local infiltration group. Although the small sample size limits definitive conclusions, this observation aligns with the hypothesis that optimal early analgesia may reduce the progression to chronic pain [2,4]. Effective RA may reduce central sensitisation, thereby preventing persistent pain.

Not all studies demonstrate long-term benefit; for instance, Albi-Feldzer et al. found no significant reduction in chronic pain with paravertebral block [26]. Differences in block technique, population characteristics, and follow-up periods may explain varying findings. In our study, pain assessment occurred at three months, which represents an early milestone; future longer-term follow-up will be needed to determine whether the absence of chronic pain persists. Nonetheless, our findings support the possibility that RA, as part of a comprehensive analgesia strategy, may reduce chronic pain development [2,5].

Enhanced functional recovery

Our findings suggest that RA facilitates faster functional recovery. A greater proportion of RA patients mobilised on the day of surgery, an important component of ERAS programmes, which aim to reduce postoperative complications such as venous thromboembolism and pulmonary issues. Better analgesia likely enabled earlier deep-breathing exercises and physiotherapy, potentially contributing to the absence of atelectasis in the RA group.

Recent evidence supports the analgesic advantages of advanced RA techniques. Santonastaso et al. demonstrated that bi-level ESP and bi-level thoracic paravertebral blocks provide effective postoperative analgesia after modified radical mastectomy, aligning with our findings that high-quality RA enhances early recovery metrics [27]. Although several non-analgesic factors (e.g., drains and sedation) may influence mobilisation, the overall trend observed in our study reinforces the recovery benefits associated with RA.

Feasibility in a resource-limited setting

A central aim of the study was to evaluate the feasibility of performing RA in a resource-limited setting. Our results demonstrate that RA was feasible in 73% of eligible cases despite limited equipment and personnel, consistent with reports from other low-resource environments where targeted training and efficient ultrasound utilisation facilitate RA implementation [10,21].

Knowledge gaps remain a significant barrier. Munasinghe et al. reported limited awareness of local anaesthetic toxicity among Sri Lankan doctors, underscoring the need for structured RA training and safety education [13]. Despite logistical constraints, patients were receptive, and block-related complications were absent, supporting RA’s safety profile in this setting. The positive outcomes observed have prompted institutional consideration of investing in additional ultrasound devices and expanding RA training, indicating that RA adoption is achievable even within limited-resource systems when backed by organisational support [28].

Comparison of block techniques

Although this study was not designed or powered to compare individual regional anaesthesia techniques, some descriptive patterns were observed. Within the regional anaesthesia group, paravertebral blocks were more commonly used for extensive procedures, whereas PECS I/II and erector spinae plane (ESP) blocks were often selected when anatomical considerations or provider preference favoured these approaches. In our cohort, none of the patients who received PECS blocks required postoperative morphine, while patients who received paravertebral or ESP blocks also had low opioid requirements overall. Given the small subgroup sizes and the absence of formal statistical comparisons or adjustment for procedure type, these findings should be interpreted as hypothesis-generating only.

Existing literature provides more robust comparative data between block types. Santonastaso et al. reported that bi-level ESP and thoracic paravertebral blocks offer effective postoperative analgesia after modified radical mastectomy [27]. Saqib et al. described improved analgesic profiles with ultrasound-guided PECS blocks compared with local infiltration [25], and ElHawary et al. found in a systematic review that ESP blocks are associated with reduced postoperative pain and opioid consumption in breast surgery [29]. Our descriptive observations are broadly consistent with these reports but do not establish comparative superiority between PECS, ESP, and PVB techniques. Larger, specifically powered randomised or comparative observational studies will be required to evaluate differential effectiveness between individual block types.

Limitations

This study has several limitations that should be considered when interpreting the findings. First, the prospective observational and non-randomised design introduces inherent risks of selection and performance bias, as the use of regional anaesthesia depended on provider availability, equipment access, and real-time workflow constraints. Blinding was not feasible and may have influenced subjective outcomes such as pain scores. Although key perioperative elements were standardised, residual confounding cannot be excluded.

Second, the planned sample size was not fully achieved due to the time-bound nature of the study and the practical realities of a resource-limited surgical oncology unit. As regional blocks became more consistently available during the study period, the majority of eligible patients appropriately received regional anaesthesia, and only 15 patients naturally fell into the infiltration group. Extending recruitment solely to complete the original target would have required intentionally withholding regional anaesthesia from additional patients, an approach that was not ethically acceptable. The reduced sample size primarily affects the precision of the effect estimates (wider uncertainty around the point estimates), particularly for secondary outcomes and block-specific comparisons, and the findings are therefore best interpreted as descriptive and hypothesis-generating.

Third, although intraoperative anaesthetic management followed a standard protocol, exact intraoperative opioid doses were not systematically quantified, and additional unmeasured factors, such as preoperative anxiety, baseline pain, surgical complexity, and postoperative adjuvant therapies, may have contributed to variability in pain scores and opioid use. PCA availability was limited to a single device on the ward; however, all postoperative opioid delivery routes (PCA, nurse-administered boluses, and infusions) were included in the 24-hour cumulative morphine calculation.

Fourth, the number of patients receiving individual block techniques (paravertebral, erector spinae plane, or PECS) was small, precluding meaningful comparative analysis between techniques. Chronic pain assessment was performed at three months, representing an early follow-up point; longer-term evaluation is needed to determine persistence of symptoms.

Finally, as a single-centre study conducted in a low- and middle-income country with significant resource constraints, generalisability to higher-resource settings may be limited. However, the study provides pragmatic, real-world evidence from an under-represented context, which remains an important strength.

Strengths

Despite these limitations, this study has several important strengths. First, it represents one of the earliest prospective evaluations of regional anaesthesia for breast cancer surgery in Sri Lanka, providing context-specific data from a low- and middle-income, resource-limited setting that is under-represented in the existing literature.

Second, data were collected prospectively using standardised anaesthetic and postoperative analgesia protocols, which reduces variability and supports internal consistency of the findings.

Third, we assessed a comprehensive range of clinically relevant outcomes, including acute pain at multiple postoperative time points, cumulative 24-hour opioid consumption, PONV, time to mobilisation, and early chronic pain, allowing a broad overview of the perioperative recovery trajectory rather than a single isolated endpoint.

Fourth, the study reflects real-world practice rather than highly controlled trial conditions, capturing the practical feasibility of implementing ultrasound-guided regional blocks within the constraints of limited equipment, staffing, and training. This pragmatic design enhances the external relevance of the findings for similar LMIC oncology centres considering wider adoption of regional anaesthesia. Finally, all eligible patients within the predefined study period were included, and analyses were conducted on an intention-to-treat basis, which helps to preserve the comparability of groups and reduce the risk of selection-related distortion, even within the constraints of an observational design.

Future directions

Future research should include randomised controlled trials to evaluate the causal impact of RA on chronic pain and recovery outcomes, and adequately powered studies comparing PECS, ESP, and PVB blocks. Economic analyses would be valuable, as RA may reduce postoperative resource utilisation despite higher initial costs. Long-term follow-up incorporating quality-of-life measures is also needed to determine whether RA prevents or delays chronic pain development. Exploring barriers to RA adoption and testing strategies to improve implementation, such as RA training programmes, is warranted. Emerging evidence on the potential influence of RA on cancer recurrence also merits exploration in future studies. Sustained institutional auditing and quality improvement efforts will be essential to integrate RA into routine breast cancer surgical care in Sri Lanka.

## Conclusions

In summary, this prospective observational study found that regional anaesthesia (RA) techniques, including paravertebral, erector spinae plane, and pectoral nerve blocks, significantly improved postoperative outcomes in breast cancer surgery within a resource-limited setting. Compared to the conventional approach of local anaesthetic infiltration, the use of RA was associated with lower acute pain scores, greatly reduced opioid consumption, a decreased incidence of postoperative nausea and vomiting, earlier mobilisation, and a markedly lower rate of chronic post-surgical pain at three months. These benefits reinforce the value of RA as a key component of multimodal analgesia for breast surgery and demonstrate that even in low-resource environments, implementing nerve block techniques is feasible and yields substantial patient advantages. Despite operational constraints, we achieved successful regional blocks in the majority of cases, with minimal additional resources (primarily the use of an ultrasound machine and skilled personnel). All three block modalities used were effective, which provides flexibility for adoption based on available expertise. Our findings should be interpreted in light of the study’s non-randomised design and sample size, but they align with a broad consensus in the literature that RA enhances recovery after mastectomy.

We recommend that centres in similar low- and middle-income country settings consider incorporating regional nerve blocks into their breast surgery protocols. The improvement in pain relief, reduction in opioid use, and potential prevention of chronic pain can significantly impact patient comfort and long-term well-being. As part of Enhanced Recovery After Surgery (ERAS) pathways for breast oncology, RA can be a game-changer even where resources are limited, as it ultimately contributes to more efficient recovery and potentially lowers the burden on healthcare systems by reducing complications and facilitating faster recovery. Ongoing efforts should focus on training, resource allocation, and research to further establish RA as a standard of care for breast cancer surgery across diverse healthcare settings.
